# The Spatial Distribution of LGR5^+^ Cells Correlates With Gastric Cancer Progression

**DOI:** 10.1371/journal.pone.0035486

**Published:** 2012-04-18

**Authors:** Eva Simon, Diana Petke, Christine Böger, Hans-Michael Behrens, Viktoria Warneke, Matthias Ebert, Christoph Röcken

**Affiliations:** 1 Institute of Pathology, Christian-Albrechts-University, Kiel, Germany; 2 Department of Medicine II, Faculty of Clinical Medicine Mannheim, University of Heidelberg, Mannheim, Germany; Enzo Life Sciences, Inc., United States of America

## Abstract

In this study we tested the prevalence, histoanatomical distribution and tumour biological significance of the Wnt target protein and cancer stem cell marker LGR5 in tumours of the human gastrointestinal tract. Differential expression of LGR5 was studied on transcriptional (real-time polymerase chain reaction) and translational level (immunohistochemistry) in malignant and corresponding non-malignant tissues of 127 patients comprising six different primary tumour sites, i.e. oesophagus, stomach, liver, pancreas, colon and rectum. The clinico-pathological significance of LGR5 expression was studied in 100 patients with gastric carcinoma (GC). Non-neoplastic tissue usually harboured only very few scattered LGR5^+^ cells. The corresponding carcinomas of the oesophagus, stomach, liver, pancreas, colon and rectum showed significantly more LGR5^+^ cells as well as significantly higher levels of LGR5-mRNA compared with the corresponding non-neoplastic tissue. Double staining experiments revealed a coexpression of LGR5 with the putative stem cell markers CD44, Musashi-1 and ADAM17. Next we tested the hypothesis that the sequential changes of gastric carcinogenesis, i.e. chronic atrophic gastritis, intestinal metaplasia and invasive carcinoma, are associated with a reallocation of the LGR5^+^ cells. Interestingly, the spatial distribution of LGR5 changed: in non-neoplastic stomach mucosa, LGR5^+^ cells were found predominantly in the mucous neck region; in intestinal metaplasia LGR5^+^ cells were localized at the crypt base, and in GC LGR5^+^ cells were present at the luminal surface, the tumour centre and the invasion front. The expression of LGR5 in the tumour centre and invasion front of GC correlated significantly with the local tumour growth (T-category) and the nodal spread (N-category). Furthermore, patients with LGR5^+^ GCs had a shorter median survival (28.0±8.6 months) than patients with LGR5^−^ GCs (54.5±6.3 months). Our results show that LGR5 is differentially expressed in gastrointestinal cancers and that the spatial histoanatomical distribution of LGR5^+^ cells has to be considered when their tumour biological significance is sought.

## Introduction

Gastrointestinal carcinomas are among the most common malignancies and are a leading cause of cancer death worldwide [1], [2]. The reasons for the poor prognoses are complex: many cancers of the gastrointestinal tract are diagnosed in advanced stages excluding curative treatment, there are no reliable tumour markers which may allow early diagnosis or screening of high-risk populations. Treatment options are limited in locally advanced and metastatic disease, and a significant number of tumours recur despite initial therapeutic response [3]. A putative explanation of an ineffective therapy is the presence of cancer stem cells (CSC). The CSC hypothesis postulates that a tumour is a conglomerate of heterogeneous cell populations. Only a subpopulation of this conglomerate maintains the capability of colony formation, and hence recurrence and metastatic spread. CSCs are more resistant to chemotherapy, leading to tumour recurrence, progression and ultimately patient death [4], [5]. While the CSC-model is increasingly accepted, identification and confirmation of so called stem cell markers in native human tissue is difficult and largely missing. Recently, the leucine-rich, G-protein coupled receptor (GPCR) and Wnt target gene *LGR5* was identified as a novel stem cell marker of the small intestine, colon and in the hair follicles of mice [6]. Expression of LGR5 in multiple other organs indicates that it may represent a global marker of adult stem cells. However, little is known about its expression in the hepato-gastrointestinal tract of humans.

In this study we aimed to fill this gap of information and tested the hypothesis that the cancer stem cell marker LGR5 has prognostic and tumour biological significance.

## Materials and Methods

### Subjects

Formalin-fixed and paraffin-embedded malignant and corresponding non-malignant tissue from 127 patients comprising six anatomical locations of the hepato-gastrointestinal tract (i.e. oesophagus, stomach, liver, pancreas, colon and rectum) were retrieved from the archives of the Institutes of Pathology of the Christian-Albrechts-University Kiel and the Charité University Hospital Berlin (both Germany). All patients were operated at either University Hospital Schleswig-Holstein (1997–2009) or Charité University Hospital Berlin (1995–2008; Table 1). Unfixed, fresh frozen tissue was available from 105 of these patients with eight different tumour types, i.e. Barrett's adenocarcinoma (9 patients) and squamous cell carcinoma (7) of the oesophagus, intestinal (19) and diffuse (21) type gastric cancer, adenocarcinoma of the colon (19) and rectum (20), hepatocellular carcinoma (HCC; 4), and cholangiocarcinoma (CC; 6). The patient characteristics are summarized in Table 1.

**Table 1:** Patient characteristics of the hepato-gastrointestinal cohort.

	Quantitative Real-time RT-PCR				Immunohistochemistry							
								TU		NT		
	Total	Mean age (range)	m∶w	*p* value*	Total	Mean age (range)	m∶w	LGR5^+^ n (%)	IRS (mean)	LGR5^+^ n (%)	IRS (mean)	*p* value^†^
**Oesophagus AC**	9	63 (51–74)	4∶5	0.007	8	64 (51–78)	5∶3	5 (63)	2.83	2 (25)	0.75	0.140
**Oesophagus SCC**	7	64 (48–73)	4∶3	0.503	6	66 (48–73)	4∶2	3 (50)	2.00	2 (33)	1.00	0.317
**Stomach intestinal**	19	69 (54–85)	8∶5	0.006	13	67 (54–85)	8∶5	12 (92)	3.54	9 (69)	2.00	0.020
**Stomach diffuse**	21	68 (43–82)	9∶10	0.013	19	69 (55–82)	9∶10	17 (90)	3.00	6 (32)	0.95	0.003
**HCC**	4	62 (43–73)	8∶8	0.545	16	59 (17–82)	8∶8	8 (50)	2.19	5 (31)	0.88	0.028
**CC**	6	63 (46–77)	4∶4	0.022	8	64 (46–77)	4∶4	6 (75)	2.38	0 (0)	0.00	0.026
**Pancreas**	0	n.d.	n.d.	n.d.	17	62 (50–78)	7∶10	17 (100)	4.29	12 (71)	2.47	0.013
**Colon**	19	70 (45–85)	9∶11	<0.001	20	74 (45–89)	9∶11	18 (90)	3.80	12 (60)	1.65	<0.001
**Rectum**	20	64 (40–84)	10∶10	0.002	20	64 (40–84)	10∶10	17 (85)	3.90	17 (85)	2.35	0.011

P values were calculated with a paired two-sided t-test (*) or the Wilcoxon test (†). Immunohistochemistry data depict the overall expression of LGR5 in malignant (TU) and corresponding non-malignant (NT) tissue. Immunoreactivity scores (IRS) were calculated for tumour cells. n.d. = not detected. AC = adenocarcinoma. SCC = squamous cell carcinoma. HCC = hepatocellular carcinoma. CC = cholangiocarcinoma. m = man. w = woman. LGR5^+^ = number (n) and percentage (%) of LGR5^+^ cases.

An independent series of 487 gastric cancer patients was retrieved from the archive of the Institute of Pathology of the Christian-Albrechts-University (Table 2 and Table 3). These patients had undergone either total or partial gastrectomy for adenocarcinomas of the stomach or oesophago-gastric junction. Each resection specimen had undergone histological examination by trained surgical pathologists. The time of patient death was obtained from the *Epidemiological Cancer Registry* of the state Schleswig-Holstein, Germany. Follow-up data of patients still alive were retrieved from hospital records and by contacting the general practitioners.

**Table 2:** Assessment of LGR5 expression in whole mount tissue sections of intestinal type gastric carcinomas.

		Luminal Surface		Tumour Centre		Invasion Front		Tumour Centre and Invasion Front	
	Total	LGR5 negative	LGR5 positive	LGR5 negative	LGR5 positive	LGR5 negative	LGR5 positive	LGR5 negative	LGR5 positive
**Patients, n (%)**	100	49 (49)	51 (51)	61 (61)	39 (39)	63 (63)	37 (37)	39 (39)	61 (61)
**Age (mean ± SD)**		69±11.8	70±9.7	69±9.9	71±11.8	69±11.3	72±9.4	68±10.4	71±10.8
**Age, n (%)**									
***p value***			0.836*		0.293*		0.200*		0.032*
<71	47	23 (49)	24 (51)	31 (66)	16 (34)	33 (70)	14 (30)	23 (49)	24 (51)
≥71	47	21 (45)	26 (55)	25 (53)	22 (47)	26 (55)	21 (45)	12 (26)	35 (74)
**Gender, n (%)**									
***p*** ** value**			0.655*		0.063*		0.648*		0.645*
Male	73	37 (51)	36 (49)	49 (67)	24 (33)	47 (64)	26 (36)	30 (41)	43 (59)
Female	27	12 (44)	15 (56)	12 (44)	15 (56)	16 (59)	11 (41)	9 (33)	18 (67)
**Localization, n (%)**									
***p*** ** value**			1.000*		1.000*		0.369*		0.129*
Proximal	32	16 (50)	16 (50)	20 (63)	12 (37)	18 (56)	14 (44)	9 (28)	23 (72)
Distal	65	33 (51)	32 (49)	40 (62)	25 (28)	44 (68)	21 (32)	29 (45)	36 (55)
**T category, n (%)**									
***p*** ** value**			0.402§		0.004§		0.889§		0.021§
T1a	6	4 (67)	2 (33)	5 (83)	1 (17)	4 (67)	2 (33)	4 (67)	2 (33)
T1b	30	14 (47)	16 (53)	21 (70)	9 (30)	20 (67)	10 (33)	15 (50)	15 (50)
T2	17	5 (29)	12 (71)	12 (71)	5 (29)	9 (53)	8 (47)	6 (35)	11 (65)
T3	32	16 (50)	16 (50)	19 (59)	13 (41)	19 (59)	13 (41)	11 (34)	21 (66)
T4a	11	6 (55)	5 (45)	3 (27)	8 (73)	7 (64)	4 (39)	2 (18)	9 (82)
T4b	4	4 (100)	0 (0)	1 (25)	3 (75)	4 (100)	0 (0)	1 (25)	3 (75)
**N category, n (%)**									
***p*** ** value**			0.352§		0.090§		0.951§		0.038§
N0	55	26 (47)	29 (53)	37 (67)	18 (33)	35 (64)	20 (36)	26 (47)	29 (53)
N1	15	6 (40)	9 (60)	11 (73)	4 (27)	10 (67)	5 (33)	7 (47)	8 (53)
N2	12	7 (58)	5 (42)	4 (33)	8 (67)	8 (67)	4 (33)	3 (25)	9 (75)
N3/a/b	16	10 (63)	6 (37)	8 (50)	8 (50)	10 (63)	6 (37)	3 (19)	13 (81)
**Stage (7th ed.), n (%)**									
***p*** ** value**			0.551§		0.046§		0.968§		0.022§
IA	30	15 (50)	15 (50)	22 (73)	8 (27)	19 (63)	11 (37)	16 (53)	14 (47)
IB	13	5 (39)	8 (62)	9 (69)	4 (31)	10 (77)	3 (23)	7 (54)	6 (42)
IIA	18	8 (44)	10 (56)	11 (61)	7 (39)	9 (50)	9 (50)	5 (28)	13 (72)
IIB	7	3 (43)	4 (57)	5 (71)	2 (29)	5 (71)	2 (29)	4 (57)	3 (43)
IIIA	10	6 (60)	4 (40)	3 (30)	7 (70)	5 (50)	5 (50)	1 (10)	9 (90)
IIIB	8	3 (38)	5 (62)	7 (88)	1 (12)	5 (63)	3 (37)	4 (50)	4 (50)
IIIC	4	3 (75)	1 (25)	0 (0)	4 (4)	3 (75)	1 (25)	0 (0)	4 (100)
IV	8	5 (63)	3 (37)	4 (50)	4 (50)	6 (75)	2 (25)	2 (25)	6 (75)
**Survival**									
***p*** ** value**			0.432£		0.467£		0.533£		0.100£
n Total	93	43	50	56	37	58	35	35	58
n Events	56	26	30	32	24	31	25	16	40
Median		34.0±6.2	48.0±13.5	44.8±9.2	33.6±9.3	39.3±9.5	34.0±10.2	54.5±6.3	28.0±8.6
95% CI		22.0–46.1	21.5–74.5	26.9–62.8	15.3–52.0	20.7–57.8	14.1–53.9	42.2–66.9	11.0–44.9

Correlation of the spatial distribution of LGR5^+^ cancer cells at the luminal surface, the tumour centre, and the invasion front with clinico-pathological patient characteristics.

* Fisher's exact test.

§ Kendall's tau.

£ log-rank test (Mantel-Cox) with a 95% confidence interval (CI). Number (n) and percentage (%) of LGR5^+^ cases. SD = standard deviation.

**Table 3:** Assessment of LGR5 expression in tissue micro arrays.

	Total	LGR5 negative	LGR5 positive	*p* value
**Patients, n (%)**	487	243 (50)	244 (50)	
**Age (mean ± SD)**		68±10.8	68±11.4	
**Age, years, n (%)**				0.635*
<65	289	144 (50)	145 (50)	
≥65	180	85 (47)	95 (53)	
**Gender, n (%)**				0.575*
Male	304	155 (51)	149 (49)	
Female	183	88 (48)	95 (52)	
**Tumour type, n (%)**				0.422*
Intestinal	189	88 (47)	101 (53)	
Diffuse	218	114 (52)	104 (48)	
Mixed	42	23 (55)	19 (45)	
**Localization, n (%)**				1.000*
Proximal	149	74 (50)	75 (50)	
Distal	315	156 (50)	159 (50)	
**T category, n (%)**				0.704*
pT1a	14	7 (50)	7 (50)	
pT1b	46	21 (46)	25 (54)	
pT2	56	23 (41)	33 (59)	
pT3	193	102 (53)	91 (47)	
pT4a	133	66 (50)	67 (50)	
pT4b	43	23 (53)	20 (47)	
**Lymph nodes, n (%)**				0.414*
No metastases	134	62 (46)	72 (54)	
Metastases	335	170 (51)	165 (49)	
**N category, n (%)**				0.884*
pN0	138	65 (47)	73 (53)	
pN1	71	38 (54)	33 (46)	
pN2	82	40 (49)	42 (51)	
pN3	69	38 (55)	31 (45)	
pN3a	71	34 (48)	37 (52)	
pN3b	50	25 (50)	25 (50)	
**Grade, n (%)**				0.088*
G1	10	6 (60)	4 (40)	
G2	103	41 (40)	62 (60)	
G3/G4	357	183 (51)	174 (49)	

Correlation of LGR5 expression with clinico-pathological patient characteristics.

* Fisher's exact test. Number (n) and percentage (%) of LGR5-positive cases. SD = standard deviation.

### Ethics Statement

All tissue samples were obtained as part of a diagnostic or therapeutic surgery carried out after the patient gave written informed consent. Patients offering samples for the study were pseudonymized and analyzed anonymously, so no individual-related data are contained in the database. The study was approved by the local ethics committee of the University Hospital in Kiel, Germany (ref. number D 453/10).

### Real-time reverse transcriptase polymerase chain reaction

Total RNA was isolated from cultured cells and cryoconserved tissues using Ambion's mirVana miRNA Isolation Kit (Applied Biosystems, Darmstadt, Germany) followed by a DNase treatment with Turbo DNA-free kit (Ambion). RNA quality was assessed in a 1.5% agarose gel. For cDNA synthesis, 2 µg of total RNA was reverse transcribed using Transcriptor First Strand cDNA Synthesis Kit (Roche Diagnostics, Mannheim, Germany). Gene-specific primers were synthesized by Biomers.net (Ulm, Germany) (Table S1). Real-time reverse transcriptase polymerase chain reaction (Real-time RT-PCR) was carried out using the LightCyler® 480 Probes Master (Roche) and the LightCycler® 480 System (Roche). The comparative Ct values were normalized to that of three housekeeping genes: *Homo sapiens succinate dehydrogenase complex, subunit A, flavoprotein (Fp) (SDHA), Homo sapiens calpain 2 (CAPN2)* and *Cyclophilin C (CYCC)*. No template controls (no cDNA in PCR) were run for each gene to detect unspecific or genomic amplification and primer dimerization. All experiments were performed in duplicates.

### Generation and purification of an anti-LGR5-antibody

Polyclonal antisera were generated against the carboxy-terminal tail of the human LGR5 receptor. Rabbits were immunized with three different peptides of the identity of SPAYPVTESCHLSSVAFVPCL (called Cterm), RSKHPSLMSINSDDVEKQSC (called 11b), CSITYDLPPSSVPSPAYPVTE (called 12) by Pineda-abservice (Berlin, Germany). Monospecific IgG was purified by fast protein liquid chromatography with ÄKTAprime™ system (GE Healthcare, Uppsala, Sweden) using a protein A-column (GE Healthcare). For all subsequent analyses, i.e. immunofluorescence, immunocytochemistry, western blot and immunohistochemistry, the monospecific IgG-fraction was affinity purified against the immunizing peptides by the Pineda-abservice. In dot blot analyses and immunohistochemical investigations, the monospecific IgG anti-LGR5-11b antibody displayed high affinity along with strong and specific immunostaining. Therefore, this antibody was used throughout this study. The reactivity and specificity of the monospecific antibody was characterized by western blot; immunofluorescence and immunocytochemical assays.

### Plasmid construct

Expression construct for LGR5 was generated by amplifying the whole coding sequence of human *LGR5* (NM_003667.2) by PCR (Table S1). To facilitate subcloning of the amplified fragment into the expression vector pcDNA3.1(−), the forward primer contained a NheI restriction site adaptor and the reverse primer contained a BamHI site and a c-Myc tag to verify successful transfection. PCR fragments and the pcDNA3.1(−) expression vector were digested separately with NheI and BamHI before ligation with T4-Ligase (Roche). The plasmid was verified by digestion with NheI and BamHI and DNA sequencing using ABI PRISM BigDye Terminator Cycle Sequencing Ready Reaction Kits, Version 2.0 (PE Applied Biosystems, Langen, Germany).

### Cell culture and transfection

The human gastric cancer cell line MKN74 was obtained from the Japanese Health Science Research Resource Bank (Osaka, Japan), and MKN45 from the German Collection of Microorganisms and Cell Cultures (Braunschweig, Germany). HEK293 EBNA cells were purchased by Invitrogen (Carlsbad, CA, USA). The cells were grown in RPMI 1640 Medium (MKN74, MKN45) or Dulbecco's Modified Eagle Medium (HEK293) supplemented with 10% (MKN74, HEK293) or 20% (MKN45) fetal bovine serum, 100 U/mL penicillin and 100 µg/mL streptomycin (PAA Laboratories GmbH, Pasching, Austria). DNA transfection of HEK293 EBNA, MKN74 and MKN45 cells was done using Lipofectamine LTX reagent (Invitrogen) according to the manufacturer's instruction. Briefly, cells were cultured in six-well plates. Stable transfection of MKN74 and MKN45 with expression plasmids was performed as previously described [7]. For transient transfection of HEK293 cells 1 µg plasmid DNA and 5 µl Lipofectamine LTX reagent were diluted in 500 µl Dulbecco's Modified Eagle Medium without serum, respectively. After incubation for 30 minutes at room temperature, the mixture was added to HEK293 cells. Forty-eight hours after transfection, transfected cells were harvested and RNA or proteins were isolated. Cells transfected with the vector pcDNA3.1(−) alone and untransfected cells served as negative controls.

### Immunofluorescence

Twenty-four hours after seeding on CultureSlides (BD Biosciences, Erembodegem, Belgium), cells were washed with phosphate-buffered saline, fixed in 7∶3 acetone∶methanol (20 minutes, −20°C) and subsequently permeabilized with 0.1% Triton-X (5 minutes, room temperature). Slides were then incubated with c-Myc mouse monoclonal antibody (Clontech, Mountain View, CA, USA) over night at 5°C in a moist chamber, followed by an anti-mouse Alexa Fluor 555 conjugated secondary antibody (Invitrogen). To detect the successful transfection of LGR5, cells were incubated with anti-LGR5-antibody followed by an anti-rabbit secondary antibody conjugated with Alexa Fluor 488 (Invitrogen) each for one hour at room temperature. Omission of primary antibodies on LGR5 transfected HEK293 EBNA cells served as negative control. Mounting and counterstaining was done with VECTASHIELD Hard-Set including DAPI (Vector Laboratories, Inc., Burlingame, CA, USA).

### Immunocytochemistry

Immunocytochemical staining was performed on formalin-fixed, paraffin-embedded MKN45 gastric cancer cells. For this purpose, MKN45 cells were embedded in small agarose beads as described previously [8]. Further processing and immunostaining with anti-LGR5-antibody (dilution 1∶1000) was carried out according to the immunohistochemical analyses of human tissue sections (see below). Omission of the primary antibody on LGR5 transfected MKN45 cells served as negative control, while the specificity of anti-LGR5-antibody staining was confirmed by incubating the antibody with the immunizing blocking peptide.

### Western blotting

Protein lysates were obtained by incubating human gastric tissue and cultured gastric cells with ProteoJET™ Mammalian Cell Lysis Reagent (Fermentas, St. Leon-Rot, Germany) and protease inhibitor cocktail (Complete EDTA-free, Roche). Protein samples were denaturated in Laemmli buffer (60 mM Tris-HCl pH 6.8, 2% sodium dodecyl sulphate, 10% glycerol, 5% β-mercaptoethanol and 0.01% bromphenol blue) by heating at 95°C for ten minutes and were subsequently loaded on 4% to 16.5% SDS polyacrylamide gels and visualized by staining with Coomassie blue. After separation, proteins on unstained polyacrylamide gels were transferred to a nitrocellulose membrane (Amersham, Freiburg, Germany), immunoblotted with the anti-LGR5-antibody (dilution 1∶20,000) and an anti-β-actin-antibody (1∶10,000; clone AC-15, Sigma-Aldrich, St. Louis, MO, USA) to ensure equal loading amounts. Membrane bound HRP labelled secondary antibodies (DakoCytomation, Glostrup, Denmark) were detected by enhanced chemiluminescence using the ECL system (Amersham). Omission of the primary antibody served as negative control, while the specificity of anti-LGR5-antibody staining was confirmed by incubating the antibody with the immunizing blocking peptide.

### Histology

For histological analyses, tissue samples were fixed in 10% neutralized formalin and embedded in paraffin. Deparaffinized sections were stained using hematoxylin and eosin (H&E). Gastric carcinoma was classified according to the WHO classification [9]. The pTNM stage was determined according to the 7^th^ edition of the International Union Against Cancer [10].

### Tissue micro array construction

Formalin-fixed and paraffin-embedded tissue samples were used to generate tissue micro arrays as described previously [11]. Four micrometer sections of the resulting tumour tissue micro array block were cut for further analysis. Successful transfer of tumour tissue was confirmed microscopically using H&E-stained sections.

### Immunohistochemistry

For immunohistochemistry, formalin-fixed and paraffin-embedded sections were used. Immunostaining was carried out with the anti-LGR5-antibody (dilution 1∶1000), a monoclonal antibody directed against CD44 (1∶200; Novocastra Laboratories Ltd, Newcastle, GB) and commercial polyclonal antibodies directed against LGR5 (LGR5^com^, 1∶400; Abcam, Inc., Cambridge, MA, USA), ADAM17 (dilution 1∶50; Sigma-Aldrich) and Musashi-1 (1∶500; Chemicon International Inc., Temecula, CA, USA). Following 20 minutes blocking with hydrogen peroxide block (Thermo Scientific, Waltham, MA, USA), 5 minutes treatment with Ultra V Block (Thermo Scientific) and incubation with the primary antibody was done in a moist chamber at room temperature for 30 minutes. Slides were washed between steps with Tris-buffered saline (TBS). Immunoreactions were visualized with the n-Histofine Simple Stain MAX PO System (Nichirei Bioscience, Tokyo, Japan) and DAB substrate (Linaris, Wertheim-Bettingen, Germany). For double staining of LGR5 with CD44, ADAM17 and Musashi-1, free binding sites were blocked with mouse-IgG and rabbit-IgG control (Abcam) diluted in antibody diluent (ZYTOMED Systems, Berlin, Germany) after DAB-treatment. The specimens were counterstained with hematoxylin. Omission of the primary antibody served as negative controls. Non-neoplastic human stomach (CD44) and brain tissue (LGR5^com^, LGR5 and Musashi-1) served as positive controls.

### Evaluation of immunostaining

Immunostaining of non-neoplastic epithelium and tumour cells was scored by applying an immunoreactivity scoring system (IRS). Briefly, category A documented the intensity of immunostaining as 0 (no immunostaining), 1 (weak), 2 (moderate), and 3 (strong). Category B documented the percentage of immunoreactive cells as 0 (negative), 1 (scattered positive cells; ≤1%), 2 (2–10% positive cells), 3 (11–50%), 4 (51–80%) and 5 (>80%). The addition of category A and B resulted in an IRS ranging from 0 to 8 for each individual case. The distributional changes of LGR5^+^ cells in 100 whole mount sections of intestinal type gastric cancer was scored as follows: the number of immunoreactive cells was categorized as 0 (negative), 1 (<10 positive cells), 2 (10–50 positive cells) and 3 (>50 positive cells) separately for the luminal surface, tumour centre and the invasion front.

### Statistical analyses

Statistical analyses were done using the PASW Statistics 18 statistical package (SPSS Inc., Chicago, IL, USA). Comparisons among groups were tested by use of Fisher's exact test. Correlation of LGR5 expression within one group was established by Kendall's tau rank-order correlation. Survival curves were fitted with the Kaplan-Meier method and differences in survival assessed by the log rank test. The significance of correlations of LGR5 expression in primary tumours and corresponding non-malignant tissue was assessed by the Wilcoxon test. Real-time RT-PCR data, which were evaluated with a paired two-sided t-test, got logarithmized to obtain approximately normally distributed data. P-values<0.05 were considered as statistically significant.

## Results

### LGR5-mRNA is differentially expressed in hepato-gastrointestinal carcinomas

LGR5 was reportedly expressed in murine stem cells of the intestinal crypts [12] and was frequently overexpressed in colon cancer cell lines [13]. To investigate its expression in non-neoplastic and neoplastic human tissue, we evaluated the transcriptional expression of LGR5 in human clinical specimens composed of a broad range of hepato-gastrointestinal carcinomas. Real-time RT-PCR analysis was carried out on a series of 105 patients comprising malignant and corresponding non-malignant tissue, obtained from the same patients, of eight different tumour types: oesophagus [Barrett's adenocarcinomas (9 patients) and squamous cell carcinomas (7)], stomach [intestinal (19) and diffuse type (21) gastric carcinomas], liver [HCCs (4), CCs (6)], colon (19) and rectum (20; Table 1).

**Figure 1:**
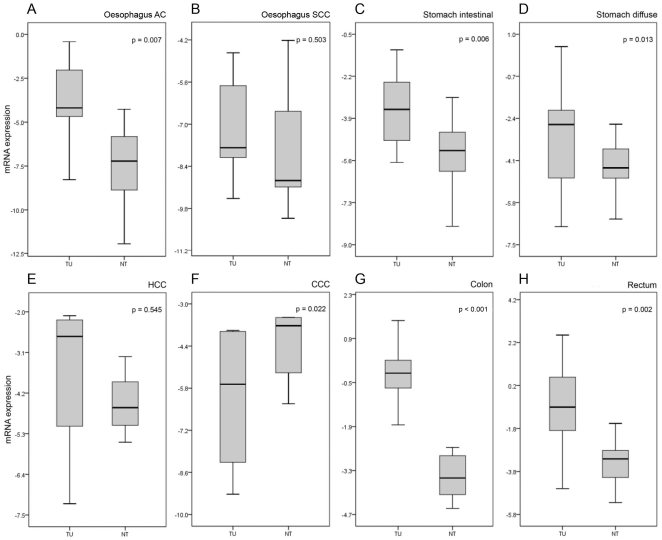
LGR5 expression in hepato-gastrointestinal tissues measured by Real-time RT-PCR. Boxplots depicting overall distribution of LGR5 comparing malignant versus adjacent non-malignant tissue in (A) Barrett's adenocarcinoma and (B) squamous cell carcinoma of the oesophagus, (C) intestinal type gastric cancer, (D) diffuse type gastric cancer, (E) hepatocellular carcinoma, (F) cholangiocarcinoma, (G) colon and (H) rectal carcinoma.

LGR5-mRNA was significantly differentially expressed in adenocarcinomas of the oesophagus (p = 0.007), stomach [intestinal type (p = 0.006) and diffuse type (p = 0.013)], liver [CCs (p = 0.022)], colon (p<0.001) as well as rectum (p = 0.002) compared with the matched non-neoplastic tissue. However, no differential expression was found in squamous cell carcinomas of the oesophagus (p = 0.503) and HCCs compared with the corresponding non-neoplastic tissues (p = 0.545; Figure 1).

### A polyclonal anti-LGR5-antibody detects human LGR5 transfected into HEK293 and MKN45 cells

**Figure 2:**
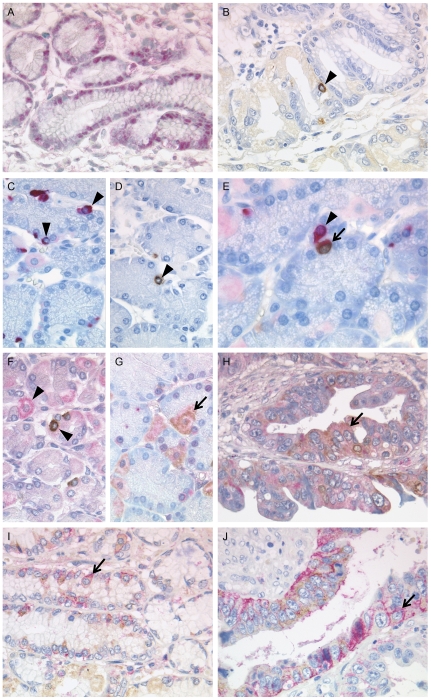
Putative stem cell marker expression in gastric specimens. Representative images of immunohistochemical staining for LGR5^com^ (A) and LGR5 (B) in a gastric cancer specimen. Following separate staining for ADAM17 (C) and LGR5 (D), as well as double staining for ADAM17 (red colour) and LGR5 (brown colour; E) in a serial section of healthy gastric mucosa. Comparing healthy stomach mucosa (F, G) and neoplastic gastric tissue (H), Msh-1^+^/LGR5^−^ (red colour), Msh-1^−^/LGR5^+^ (brown colour; F) as well as Msh-1^+^/LGR5^+^ cells (G) are present. Predominantly CD44^+^/LGR5^+^ cells in double staining experiments for CD44 (red colour) and LGR5 (brown colour) in healthy (I) and neoplastic (J) gastric mucosa. Double positive cells are indicated by arrows, arrowheads mark scattered single stained cells. Original magnifications ×400 (A–J); ×600 (E).

**Figure 3:**
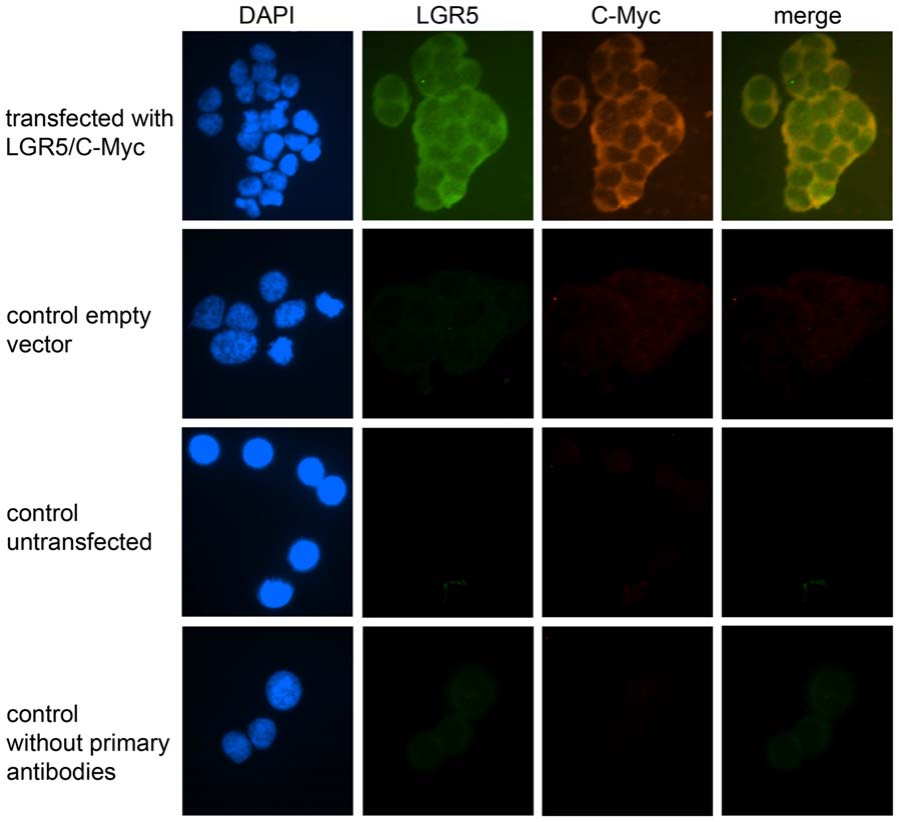
Immunofluorescence staining with anti-LGR5-antibody. Immunofluorescence staining of HEK293 EBNA cells with LGR5 specific antibody (green) and anti-myc tag antibody (red). Cells were counterstained with DAPI to show the cell nucleus (blue). Cells transfected with the myc-tagged LGR5 cDNA (first panel) compared to control cells, transfected with the empty vector (control empty vector); leaving untransfected (control untransfected); or incubated without the primary antibodies , respectively. Original magnifications ×400.

To further explore the histoanatomical distribution of LGR5 in human tissues and to investigate its clinico-pathological significance, an LGR5-specific antibody was raised, since the commercially available antibodies did not meet our demands (Figure 2A). To exclude cross reactivity of the newly generated antibody with the most structurally similar LGRs, LGR4 and LGR6, we performed a sequence alignment with the *National Center for Biotechnology Information* alignment tool in advance (data not shown). No sequence homology was found with LGR4 or LGR6 in the region of the LGR5 peptide we used for immunization. For the selection of a highly specific antibody against LGR5, cloned full length cDNA was transfected into HEK293 EBNA cells and used as a positive target performing immunofluorescence. The LGR5 cDNA was directly tagged with a myc-epitope, which enabled the evaluation of the transfection with an anti-myc tag antibody. Using immunofluorescence, binding of the anti-myc tag antibody was found after incubation with LGR5/HEK293 cells, but not in empty vector-transfected cells (vector/HEK293), untransfected HEK293 cells, or in the negative control of LGR5/HEK293 cells incubated with antibody diluent instead of the primary antibody. The same result was obtained when we used the anti-LGR5-antibody (Figure 3), demonstrating specific labelling of LGR5.

The specificity of LGR5-immunolabelling was further validated using formalin-fixed and paraffin-embedded MKN45 gastric cancer cells. The cells were prepared and stained in a manner that parallels the processing of clinical human tissue specimens. Immunocytochemical analyses on paraffin sections revealed a positive staining of anti-LGR5-antibody on MKN45 cells stably transfected with LGR5 cDNA (LGR5/MKN45). Instead, empty vector-transfected MKN45 cells (vector/MKN45), as well as the negative control (omission of the primary antibody) and the peptide control (pre-incubation of the antibody with its immunizing blocking peptide) of LGR5/MKN45 cells showed no binding of the antibody (Figure S1).

### LGR5 protein is specifically detected by anti-LGR5-antibody in human transfected MKN74 cells

The human gastric cancer cell lines MKN74, known to posses a moderate LGR5-mRNA expression (data not shown) and human non-neoplastic stomach, exhibiting very low LGR5 expression (Figure 1) were chosen to characterize the reactivity and specificity of the anti-LGR5-antibody on protein level.

**Figure 4:**
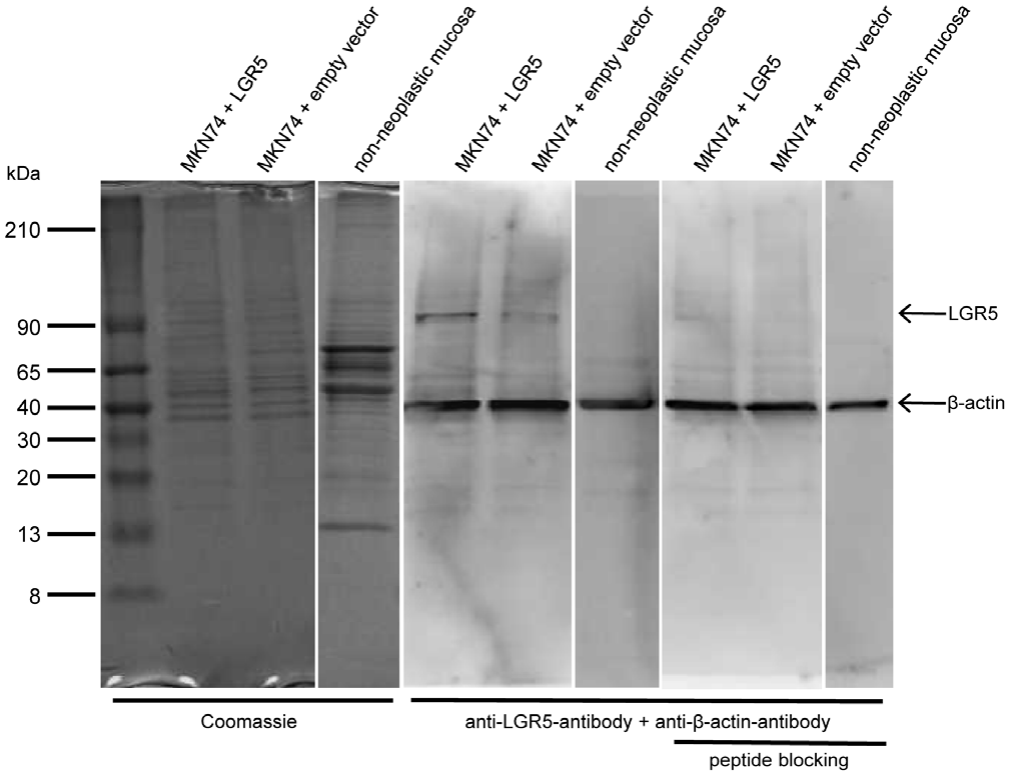
Validation of the anti-LGR5-antibody by western blotting. Coomassie blue staining (Coomassie) depicts the quality of loaded total protein lysates. In western blot analysis the anti-LGR5-antibody (1∶20,000) detects a single band in protein lysates of stably transfected MKN74 cells, overexpressing LGR5 (MKN74+LGR5), a weaker band of cells transfected with the control empty vector (MKN74 + empty vector), and no band in lysates of human non-neoplastic stomach mucosa, respectively. On a parallel blot no target bands are visible when the anti-LGR5-antibody was pre-incubated with its immunizing blocking peptide (peptide blocking). The top bands (100 kDa, arrow) display the LGR5 protein, whereas the bottom bands (∼43 kDa, arrow) depict β-actin, used as a loading control.

In western blot analysis, LGR5 protein was recognized specifically by the monospecific anti-LGR5-antibody at a dilution of 1∶20,000. It identified one band with an apparent molecular weight of 100 kDa in MKN74 cell lysates (Figure 4), which matches the molecular mass calculated for LGR5 protein. In contrast, no band was detected in lysates of human non-neoplastic stomach tissue, which excludes a cross reactivity of anti-LGR5-antibody with cellular proteins. In line with mRNA expression data, stronger expression of LGR5 protein was observed in MKN74 cells stably transfected with LGR5 cDNA, compared to MKN74 control cells transfected with the empty vector. LGR5 protein expression in human non-neoplastic stomach mucosa was beyond the detection limit of western blotting, displaying no band. Omission of the anti-LGR5-antibody or previous incubation of the antibody with its immunizing blocking peptide displays no protein bands, respectively. Detection of β-actin protein (about 43 kDa; Figure 4) served as a loading control and confirmed evenly loaded protein amounts in all lanes.

### LGR5 is expressed in non-neoplastic and neoplastic tissue of the hepato-gastrointestinal tract

The expression and histoanatomical distribution of LGR5 was subsequently studied by immunohistochemistry in a series of 127 tissue slides obtained from corresponding non-neoplastic and neoplastic tissue of the hepato-gastrointestinal tract, comprising the oesophagus (14 patients), stomach (32), liver (24), pancreas (17), colon (20), and rectum (20; Table 1). Out of this cohort neoplastic and corresponding non-neoplastic tissue samples of 105 patients were also studied on the transcriptional level (see above).

**Figure 5:**
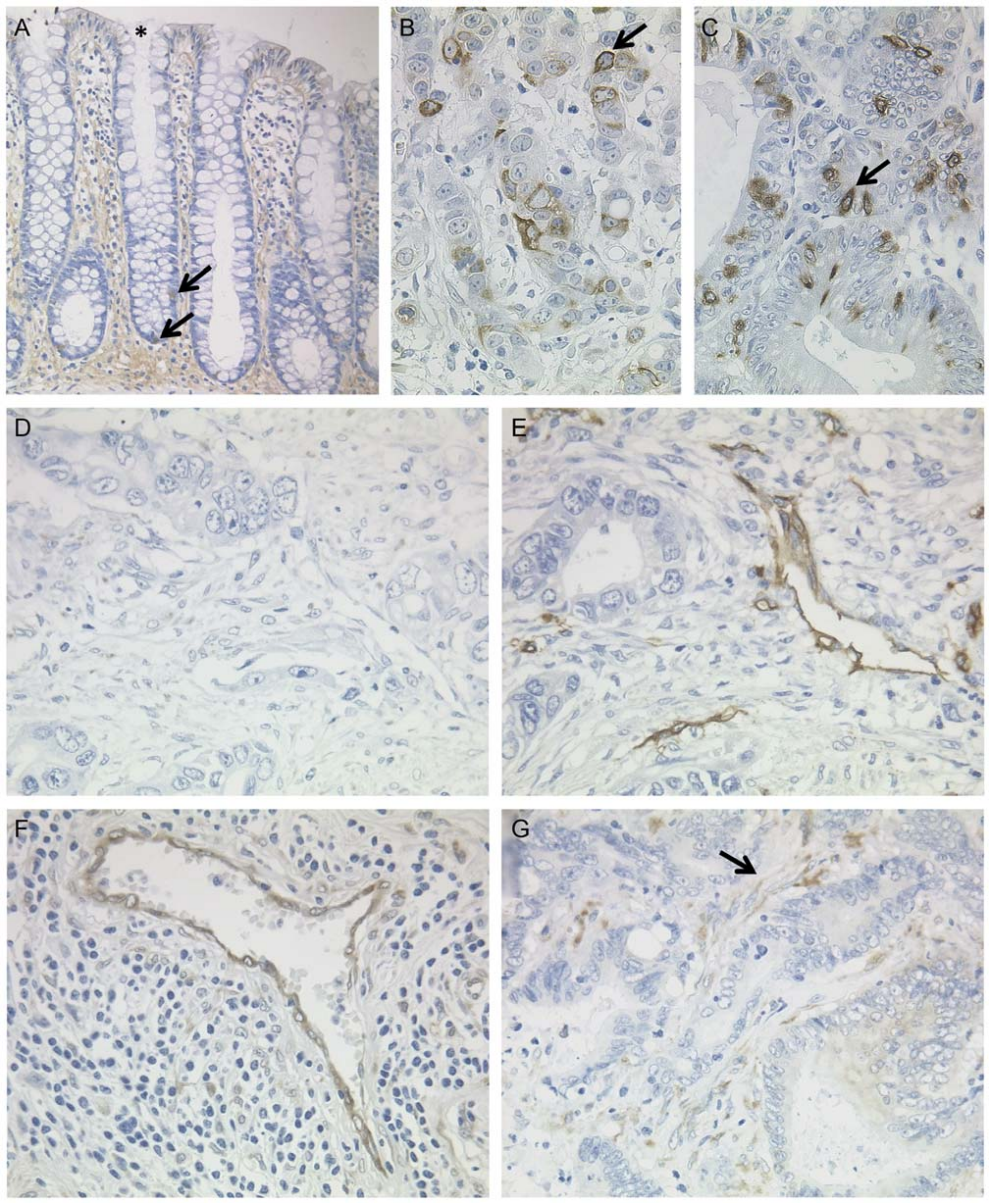
Immunohistochemical staining of LGR5 in colon tissues. LGR5 expression in normal colonic mucosa (A), membranous (B) and cytoplasmic (C) staining in corresponding colon cancer cells. Arrows mark scattered immunoreactive cells within the crypt base. Asterisk (*) marks the luminal site. Variable LGR5-immunoreactivity of endothelial cells was found in cancer tissue: The presence of LGR5-immunonegative endothelial cells (D) was confirmed by CD34 (E) using serial sections. Note strong endothelial LGR5-immunoreactivity in another case of colon cancer (F). (G) Expression of LGR5 in desmoplastic stroma cells. Original magnifications ×200 (A); ×400 (B–G).

LGR5 expression of the overall cohort was positive in 65 cases (51%) of all non-malignant tissues and 103 cases (81%) of all cancer tissues. Localization of LGR5 expression was observed as mainly cytoplasmatic, whereas also a sporadic membrane or core membrane accentuated expression occurred. An LGR5-immunoreactivity was found in all tissue components, i.e. stroma cells, endothelial cells, cells in the non-neoplastic epithelium and in cancer cells (Figure 5). LGR5-immunoreactivity in stroma cells was observed in 49 (39%) of all non-malignant tissues and in 80 cases (63%) of all cancer tissues. An endothelial immunoreactivity of LGR5 was observed in 42 cases (33%) of all tissues.

**Figure 6:**
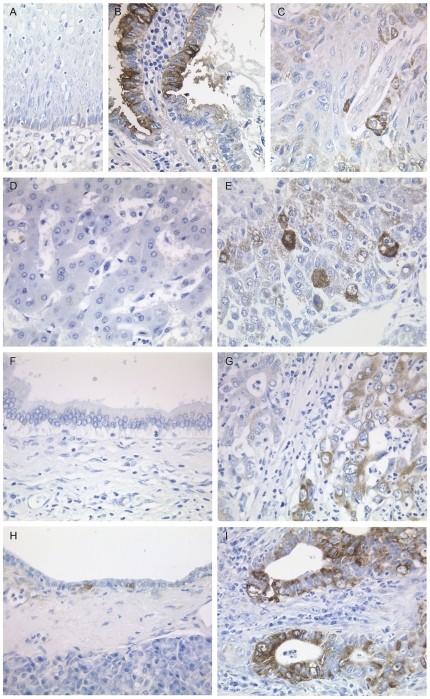
LGR5-immunoreactivity in hepato-gastrointestinal tissues. Expression of LGR5 in normal oesophageal mucosa (A) compared with an adenocarcinoma (B) and a squamous cell carcinoma (C). LGR5 expression in the normal liver (D) compared with hepatocellular carcinoma (E), normal (F) and malignant (G) epithelium of the bile duct, as well as non-neoplastic (H) and neoplastic (I) pancreatic tissue. Original magnifications ×400.

In the non-malignant epithelium, LGR5 was usually found in few scattered cells close to the basement membrane of the gastric mucosal unit, pancreatic ducts, and the colorectal crypts. LGR5 was not found in the squamous epithelium of the oesophagus, intrahepatic bile ducts, and hepatocytes (Figure 6). For all tissue types apart from oesophageal tissue the mean value of IRS was significantly higher for tumour tissue relative to the matched non-malignant tissue, confirming our findings on the transcriptional level (Table 1).

### LGR5 is present in scattered cells of the normal gastric mucosa

**Figure 7:**
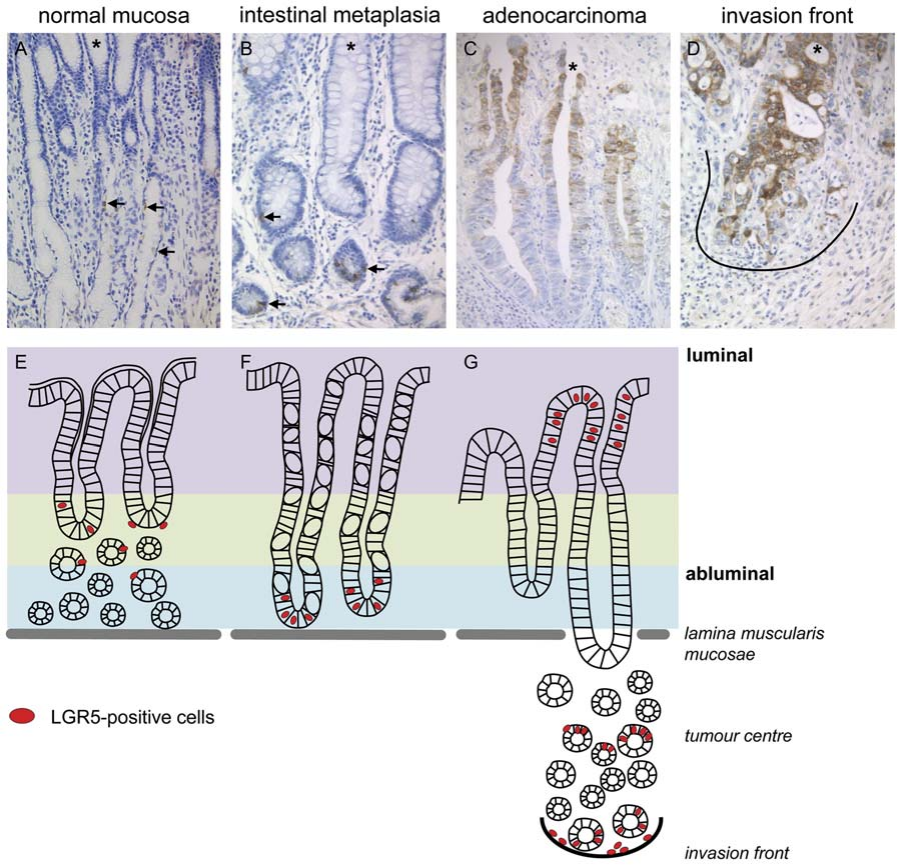
Staining patterns of LGR5 in gastric cancer tissues. Distribution patterns of LGR5^+^ cells in healthy gastric mucosa (A, E), an intestinal metaplasia (B, F), and a gastric adenocarcinoma (C, G) with its invasive front (D). The upper panel shows representative immunohistochemical staining with an anti-LGR5-antibody on whole mount sections of intestinal type gastric cancers. The lower panel depicts the corresponding schematic model of the distributional changes in different stages of gastric tumourigenesis. Arrows mark LGR5^+^ cells. Asterisk (*) marks the luminal site. The black line highlights the tumour host interface (invasion front). Original magnifications ×200.

The existence of multipotent stem cells within the murine stomach was demonstrated by clonal marking studies. The definitive identification of these cells failed so far owing the unavailability of specific endogenous markers [14]. Using serial sections obtained from a sleeve resection specimen, which is usually obtained for the treatment of overweight, and showed no histological evidence of gastric cancer or chronic gastritis, we searched for LGR5^+^ epithelial cells. Interestingly, scattered LGR5^+^ cells were found in the mucous neck region between the foveolae and glands (Figure 7).

### LGR5 is coexpressed with other potential gastric stem cell or progenitor cell markers

Stem cells of non-neoplastic tissue and CSCs are usually identified and validated by the expression of more than a single stem cell marker [15]. Using immunohistochemistry, we next analyzed the coexpression of LGR5 with other putative CSC markers, i.e. ADAM17, CD44, Musashi-1 [16]–[21]. ADAM17 was selected, since previous studies identified ADAM17 as a putative stem cell marker of the stomach [8]. Immunostaining (either double staining or staining of serial sections) was carried out on a gastric cancer specimen and on healthy uninflammed gastric mucosa obtained by sleeve gastrectomy. In general only few scattered cells showed positive immunostaining for one and/or the other putative stem cell marker. Interestingly, the non-neoplastic gastric mucosa harbours cells which are each ADAM17^+^/LGR5^+^ (representing approximately one third of all immunoreactive cells) and ADAM17^+^/LGR5^−^ (two thirds), and ADAM17^−^/LGR5^+^ (few scattered cells; Figure 2C–E).

Musashi-1 (Msh-1) was described as a putative stem cell marker in the mouse intestine and human stomach [18], [22] and as a CSC marker in tumours [23]. In neoplastic and non-neoplastic gastric tissue positive stained cells composed predominantly of Msh-1^+^/LGR5^−^ (approximately two thirds of all immunoreactive cells), to a lesser extent of Msh-1^+^/LGR5^+^ (one third), and few Msh-1^−^/LGR5^+^ cells (Figure 2F–H). Finally the surface marker of colorectal and gastric cancer stem cells CD44 [21], [24] revealed a distribution comparable with Msh-1 (Figure 2I, J). Collectively, these findings support the hypothesis that our anti-LGR5-antibody detects cells with stem cell expression patterns in the human gastric mucosa.

### The spatial distribution of LGR5^+^ cells changes during tumourigenesis

The sequential changes in the gastric mucosa that precede the development of invasive cancer are known as the ‘precancerous cascade’, first described in 1975, where normal gastric mucosa is transformed by chronic atrophic gastritis and develops multifocal atrophy and intestinal metaplasia, followed by the appearance of dysplasia and finally invasive carcinoma [25]. Next we tested the hypothesis that the aforementioned histopathological changes of the stomach mucosa are associated with a reallocation of the LGR5^+^ putative stem cells. We systematically explored the histoanatomical distribution of LGR5^+^ cells in 100 patients with intestinal type gastric cancer. Whole mount tissue sections were used which enclosed non-neoplastic, metaplastic and neoplastic cancer tissue. It was readily apparent that the histoanatomical distribution of LGR5^+^ cells changed (see Figure 7): in non-neoplastic and non-metaplastic stomach mucosa, few scattered LGR5^+^ cells were found in the mucous neck region (Figure 7A, E). In the intestinal metaplasia, a slightly increased number of LGR5^+^ cells were localized at the bottom of the metaplastic crypts (Figure 7B, F). However, in intestinal type gastric cancer the localization and number of LGR5^+^ cells was strikingly changed. In gastric cancer, LGR5^+^ cells were present at the luminal surface (Figure 7C, G), in the tumour centre (between luminal surface and invasion front) and at the invasion front (Figure 7D). Most interestingly, the distribution of LGR5^+^ gastric cancer cells showed distinctive patterns: the LGR5^+^ cells occurred in cohesive patches of a variable number of tumour cells ranging from <10, 10–50 and even >50 tumour cells, often forming gradients of decreasing staining intensities. The overall distribution of these LGR5^+^ tumour cell patches was uneven and inhomogeneous. Collectively, we observed an increase in the number and intensity of LGR5^+^ cells from non-neoplastic epithelia to gastric cancer supporting our Real-time RT-PCR and immunohistochemistry data of matched (non-neoplastic versus neoplastic) patient cases (see Table 1). Our findings of the altered distribution pattern of LGR5^+^ cells is consistent with those described by Takeda et al. for colorectal cancer [26].

### The expression of LGR5 in intestinal type gastric cancer correlates with local tumour growth and nodal spread

**Figure 8:**
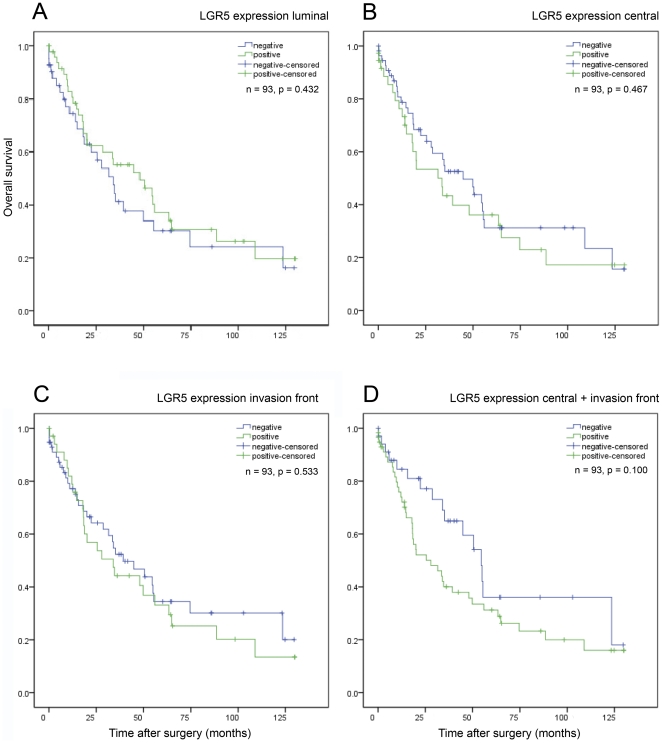
Survival of the validation cohort. Kaplan-Meier curves depicting overall survival of the validation cohort according to the distribution of LGR5^+^ gastric cancer cells at the luminal surface (A), the tumour centre (B), and the invasion front (C). (D) Survival of grouped patients with LGR5^+^ tumour cells in the tumour centre and/or at the invasion front. P-values were calculated with the log rank test.

It is postulated that CSC influence patient prognosis by their ability to form tumour cell colonies, an indispensable pre-requisite for metastatic spread and disease recurrence. To further explore the significance of LGR5 for gastric cancer, we correlated the expression of LGR5 in intestinal type gastric cancer with various clinico-pathological patient characteristics. Since the spatial distribution of LGR5^+^ cancer cells and hence the putative stem cell niche may be important for its tumour biological significance, we investigated each compartment, i.e. luminal surface, tumour centre and invasion front, separately. This showed a significant correlation between the presence of LGR5^+^ cancer cells in the tumour centre and the local tumour growth (T-category; Table 2). A detailed analysis of the patient survival further revealed that patients with LGR5^+^ cancer cells at the luminal surface lived longer (compared with LGR5-negative cases at the luminal surface), while those with LGR5^+^ tumour cells in the tumour centre and at the invasion front lived shorter compared with LGR5-negative cases at these sites (Table 2; Figure 8). This indicated that the biological significance of LGR5^+^ cancer cells may depend on their spatial distribution within the tumour mass. In the next step we grouped all patients together with LGR5^+^ tumour cells in the tumour centre and/or at the invasion front. This now showed that LGR5 expression correlated significantly with the local tumour growth (T-category), nodal spread (N-category) and tumour stage. Furthermore, patients with LGR5^+^ tumour cells had a shorter median survival (28.0±8.6 months) compared with LGR5-negative cancers (54.5±6.3 months; Figure 8). Although, this difference did not reach statistical significance, it was interesting to note that the confidence intervals of LGR5-positive and -negative cases barely overlapped (Table 2; Figure 8).

### Tissue micro arrays are not applicable to study the tumour biological significance of LGR5

Tissue micro arrays (TMA) are commonly used to study the tumour biological significance of novel biomarkers [27]. However, as shown above, the distribution of LGR5^+^ cells is not random and the tumour biological effect depends on their spatial distribution. Finally we wished to test, whether TMAs are suitable to study the tumour biological significance of putative CSC markers. We constructed TMAs from a series of 487 patients, who underwent partial or complete gastrectomy for adenocarcinomas of the oesophago-gastric junction or stomach. Survival data were available from 466 patients. 333 patients died during follow-up and 51 were still alive at the endpoint of our analysis. The median follow-up for those patients still alive was 60.9 months (range 14.3–129.9 months).

LGR5^+^ tumour cells were found in 244 of 487 (50%) patients. A strong cytoplasmic and membranous immunoreaction was observed in 36 (7.4%) cases and a weak to moderate staining in 208 (42.7%) cases. 243 tumours (50%) lacked LGR5-immunoreactivity (Table 3). Next we studied the correlation between the expression of LGR5 and various clinico-pathological patient characteristics. The expression of LGR5 by tumour cells did not correlate with any clinico-pathological patient characteristic, including patient survival (Table 3). However, patient survival correlated significantly with tumour type (p = 0.007), T-category (p<0.001), the presence of lymph node metastases (p<0.001), N-category (p<0.001) and tumour grade (p<0.001; Table 4).

**Table 4:** Patient survival related to several clinico-pathological patient characteristics in tissue micro arrays.

	Patients	Events	Median survival, months (95% Cl)	Log rank test
**Overall survival**	466	333	15.5 (13.1–17.9)	
**Age, years**				0.186
<65	288	210	13.6±1.5 (10.7–16.5)	
≥65	178	123	16.6±2.2 (12.4–20.8)	
**Tumour type**				0.007
Intestinal	181	124	19.5±3.1 (13.4–25.6)	
Diffuse	209	154	13.2±1.3 (10.8–15.7)	
Mixed	41	32	10.6±1.9 (7.0–14.3)	
**Localization**				0.062
Proximal	147	113	12.6±1.0 (10.7–14.6)	
Distal	298	205	17.9±2.0 (13.9–22.0)	
**T category**				<0.001
pT1a	14	2	123.5±49.7 (26.1–220.9)	
pT1b	44	16	64.9	
pT2	50	28	35.2±10.3 (14.9–55.4)	
pT3	186	137	16.7±2.0 (12.8–20.6)	
pT4a	128	109	9.8±1.9 (6.1–13.4)	
pT4b	43	40	9.3±1.6 (6.1–12.5)	
**Lymph node metastases**				<0.001
No metastases	128	52	55.0±15.8 (24.0–86.0)	
Metastases	322	270	11.9±1.0 (9.9–13.9)	
**N category**				<0.001
pN0	132	53	55.5±18.7 (18.9–92.0)	
pN1	67	46	20.0±3.7 (12.9–27.2)	
pN2	78	63	16.6±2.0 (12.8–20.5)	
pN3	68	60	8.3±1.2 (5.9–10.8)	
pN3a	69	61	9.4±1.9 (5.5–13.2)	
pN3b	48	46	6.4±1.7 (3.0–9.8)	
**Grade**				<0.001
G1	10	4	56.0±21.0 (14.8–97.0)	
G2	104	60	34.0±8.1 (18.1–49.8)	
G3/G4	340	260	12.7±0.8 (11.1–14.3)	
**LGR5 at tumour centre and/or invasion front**				0.100
LGR5-positive tumour cells	58	40	28.0±8.6 (11.0–44.9)	
LGR5-negative tumour cells	35	16	54.5±6.3 (42.2–66.9)	
**LGR5 expression assessed by tissue micro array technology**				0.915
LGR5-positive tumour cells	237	172	15.5±1.5 (12.6–18.3)	
LGR5-negative tumour cells	228	160	14.7±1.5 (11.7–17.7)	

Patient survival according to several clinico-pathological parameters and the expression of LGR5 in tumour cells assessed in tissue micro arrays and whole mount tissue sections, respectively. P-values were calculated with the log rank test (Mantel-Cox) with a 95% confidence interval (CI).

## Discussion

Carcinomas of the hepato-gastrointestinal tract still head the statistics of cancer deaths worldwide [28]. There is a need for new molecular-targeted therapeutic approaches in order to improve the poor prognosis of hepato-gastrointestinal cancers. Targeting cancer stem cells (CSCs) might be a novel approach to improve patient outcome. CSCs like normal tissue stem cells (SCs) are capable of self-renewal, asymmetric division and multilineage differentiation enabling them to efficiently seed new tumours upon inoculation into recipient hosts, such as mice [29], [30]. They are likely to depend on a stem cell niche and the expression of stem cell markers is influenced and modulated by the local cellular and acellular environment [31]. The identification of stem cell markers, and the characterization and validation of CSC for solid human tumours remains a major obstacle. The validity of the CSC hypothesis for solid human tumours can only be confirmed circumstantially.

Recently, Barker and colleagues used lineage labelling to show that LGR5 expressing cells represent actively dividing multipotent stem cells of the gastric and intestinal mucosa of mice [6]. Based on this observations we hypothesized that LGR5, may have an impact on tumourigenesis in the human gastrointestinal tract.

Using a monospecific anti-LGR5-antibody, we were able to identify LGR5 expressing cells in non-neoplastic and neoplastic tissue. Few, scattered LGR5^+^ cells were found in close proximity to the basement membrane of non-neoplastic pancreatic ducts, the colorectal crypts, and the stomach. All these sites are compatible with the histoanatomical locations of stem cells in humans [6], [26], [32]. LGR5 expressing cells at the base of antral glands represent actively dividing multipotent stem cells that can give rise to all antral unit cells in the mouse [6]. Similarly, we show that LGR5 is expressed by cells at the base of the human gastric glandular unit, which may represent those stem cells described by Barker and colleagues in the murine stomach.

Inspired by the work of Baker et al. other researchers continued to characterize the Wnt target gene and G-protein coupled receptor in mice [12], [33], [34], human cell cultures [16], [35] and cancer specimens [13], [24], [36]. After all LGR5 is currently considered to be the most selective and promising marker of SCs and CSCs in the intestinal epithelium [16]. However, a systematic analysis of LGR5 expression in a broad range of human hepato-gastrointestinal tumours and their corresponding normal tissue was as yet missing. With our experimental approach, we provide evidence that LGR5 is significantly up-regulated in a large variety of human hepato-gastrointestinal cancers relative to the tumour adjacent normal tissue. In support of this contention, the expression of LGR5 in intestinal type gastric cancer correlated with the local tumour growth, nodal spread (as an indicator for the ability of colony formation) and patient survival. Nonetheless, whether LGR5 up-regulation itself contributes to cancer progression or simply is a surrogate marker, necessitates further investigations.

As a matter of course, LGR5 is not the only marker of stemness in the stomach. Several markers including ADAM17 [8], CD44 [19], and Musashi-1 [37] were reported as putative markers of SC or CSC in the gastrointestinal tract. The specificity of our anti-LGR5-antibody was initially confirmed by western blotting and immunofluorescence staining. The apparent coexpression and colocalization of above-mentioned putative stem cell markers and LGR5 in double staining experiments assures our conjecture that the LGR5-antibody identifies those repeatedly described LGR5^+^ SCs and CSCs in human tissues. After all the expression of LGR5 and other putative stem cell markers appear to be early events and to remain high in neoplastic gastric tissue.

The assumption that LGR5 is a stem cell marker of both non-neoplastic and neoplastic gastric epithelia raises several interesting questions. LGR5 is a member of the canonical Wnt-signalling cascade, which forms a signalling gradient in the intestinal crypt and thereby regulates cell proliferation and differentiation [38]. LGR5 highlights the stem cell niche and loss of LGR5 expression indicates the growth direction of the descending cells [14]. As demonstrated by our immunohistochemical studies, the number and histoanatomical distribution of LGR5^+^ cells is variable. In the non-neoplastic mucosa, the stem cells differentiate into two directions, i.e. towards the luminal surface forming the foveolar epithelium, and into the abluminal compartment forming gastric glands [14]. This bidirectional differentiation seems to be lost in intestinal metaplasia, where LGR5^+^ cells were present at the metaplastic crypt base. The direction of cell differentiation must have changed, now pointing only towards the luminal surface. While a gradient of LGR5 expression was hardly detectable in non-neoplastic mucosa, gastric cancer frequently showed a gradient of LGR5 expression. Most surprisingly, a very strong expression of LGR5 was found at the luminal surface of gastric cancer with decreasing staining intensities of the adjacent abluminal cells. This observation leads to the contention that the putative stem cell niche has become far more mobile by obtaining migratory capabilities and less restricted with regard to the spatial orientation of its derivatives, which could now also proliferate towards the abluminal site of the mucosa, supporting an invasive growth direction. Furthermore, the overall expression pattern of LGR5 in gastric cancer is compatible with the loss of control of the growth direction of neoplastic epithelia. The uneven distribution of CSC and the seemingly “uncontrolled” or “chaotic” growth direction of their derivatives may form the fundamental basis of cancer morphology, which is used ultimately for the histopathological classification of malignant tumours.

Our observations emphasizes the necessity to consider the histoanatomic distribution of LGR5^+^ cells in tissue based studies, since the tumour biological effect is clearly related to their spatial distribution. This is mostly ignored by current studies, running the risk of misinterpreting obtained results, leading to questionable conclusions. Our hitherto unreported finding in gastric carcinoma indicates that especially TMA are not applicable to study the tumour biological significance of LGR5.

Nevertheless, our findings are in line with current studies, identifying LGR5 expressing cells as the cells of origin in intestinal tumours [39]. LGR5 as an optional Wnt coreceptor, is assumed to mediate the enhancement of Wnt signals by binding soluble R-spondin proteins [40], [41], therefore playing a role in tumour growth and metastasis [13], [42], [43]. However, Walker and colleagues reported that LGR5 suppression in colorectal cancer cell lines induced increased invasion, growth and enhanced tumourigenicity. They conclude that LGR5 may be important in restricting stem cells to their niche and loss of LGR5 may contribute to the invasive phenotype of colorectal carcinoma [16]. In addition to that, our findings now implicate that LGR5^+^ cells and hence the number of stem cells increases and that the stem cell niche re-allocates during carcinogenesis, i.e. to the luminal surface, tumour centre and invasion front. LGR5^+^ cells lose their restriction to the stem cell niche of the non-neoplastic mucosa. These observations are supported by Takeda et al. [26]. Notably the LGR5 expression at the tumour centre and the invasion front correlated with tumour growth and nodal spread.

A problem associated with characterization of CSC of solid cancer is their separation from the surrounding cell population [44]. The native tumour microenvironment is likely to have a profound influence on the tumourigenic process [45], also contributing to the maintenance of the stemness of stem cells [40]. Even the use of tumour cell lines carries the risk of providing misleading results.

In conclusion, we provide evidence for an increase of LGR5^+^ putative stem cells during gastric tumourigenesis and that the reallocation of stem cells, e.g. towards the tumour centre and invasion front, may play a role in the development and progression of gastric cancer. The spatial histoanatomical distribution of LGR5^+^ cells has to be considered, when their tumour biological significance is explored in future studies. A more broadly applicable biomarker that facilitates the identification and characterization of CSC populations in different tumour sites is essential, enabling the development of more effective cancer therapies [46]. LGR5 seems to be a more general marker of stemness in the gastrointestinal tract, helping to raise novel hypothesis for the involvement of CSC in tumour development, progression and growth patterns. However, its tumour biological function remains obscure and necessitates further investigations.

## Supporting Information

Table S1 **Primer sequences for polymerase chain reaction.**
(DOC)

Figure S1 **Immunocytochemistry of stably transfected MKN45 cells.** LGR5-immunoreactivity in MKN45 gastric cancer cells stably transfected with LGR5 cDNA (LGR5/MKN45) (A) compared to control cells, transfected with the empty vector (B). The lower panel depicts LGR5/MKN45 cells incubated without the primary antibody (C) or after pre-incubation of anti-LGR5-antibody with its immunizing blocking peptide, respectively (D). Original magnifications ×600.
(TIF)
