# Supplementary material for: The Spatial Distribution of LGR5+ Cells Correlates With Gastric Cancer Progression
Source: PLoS One. 2012 Apr 18;7(4):e35486. doi: 10.1371/journal.pone.0035486 (PMC3329462; doi:10.1371/journal.pone.0035486)
Supplement: Table S1 — Primer sequences for polymerase chain reaction. (DOC) [file pone.0035486.s001.doc]

**Table S1.** PCR primer sequences

| **Gene symbol** | **Forward primer 5’->3’** | **Reverse primer 5’->3’** |
| --- | --- | --- |
| *LGR5* | AAT TTG CGA AGC CTT CAA TC | GGG ATT TCT GTT AAC GCA TTG |
| **Reference genes** |  |  |
| *SDHA* | ATT TGG TGG ACA GAG CCT CA | CTG GTA TCA TAT CGC AGA GAC CT |
| *CAPN2* | CGC TGA CCC CCA GTT TAT C | TCA AGG TGA GGG AGG CAA T |
| *CYCC* | GGA AAA GTC ATT GAT GGG ATG | CAA AAG GCG TTT TCA CGT CTA |
| **Cloning primers** |  |  |
| *LGR5* | CGC GCT AGC ATG GAC ACC TCC CGG CTC GG | CGC GGA TCC CTA CAG ATC TTC TTC AGA AAT­AAG TTT TTG TTC GAG ACA ­TG­G GAC AAA TGC CA |
