# Supplementary figures and images for: The Spatial Distribution of LGR5+ Cells Correlates With Gastric Cancer Progression
Source: PLoS One. 2012 Apr 18;7(4):e35486. doi: 10.1371/journal.pone.0035486 (PMC3329462; doi:10.1371/journal.pone.0035486)

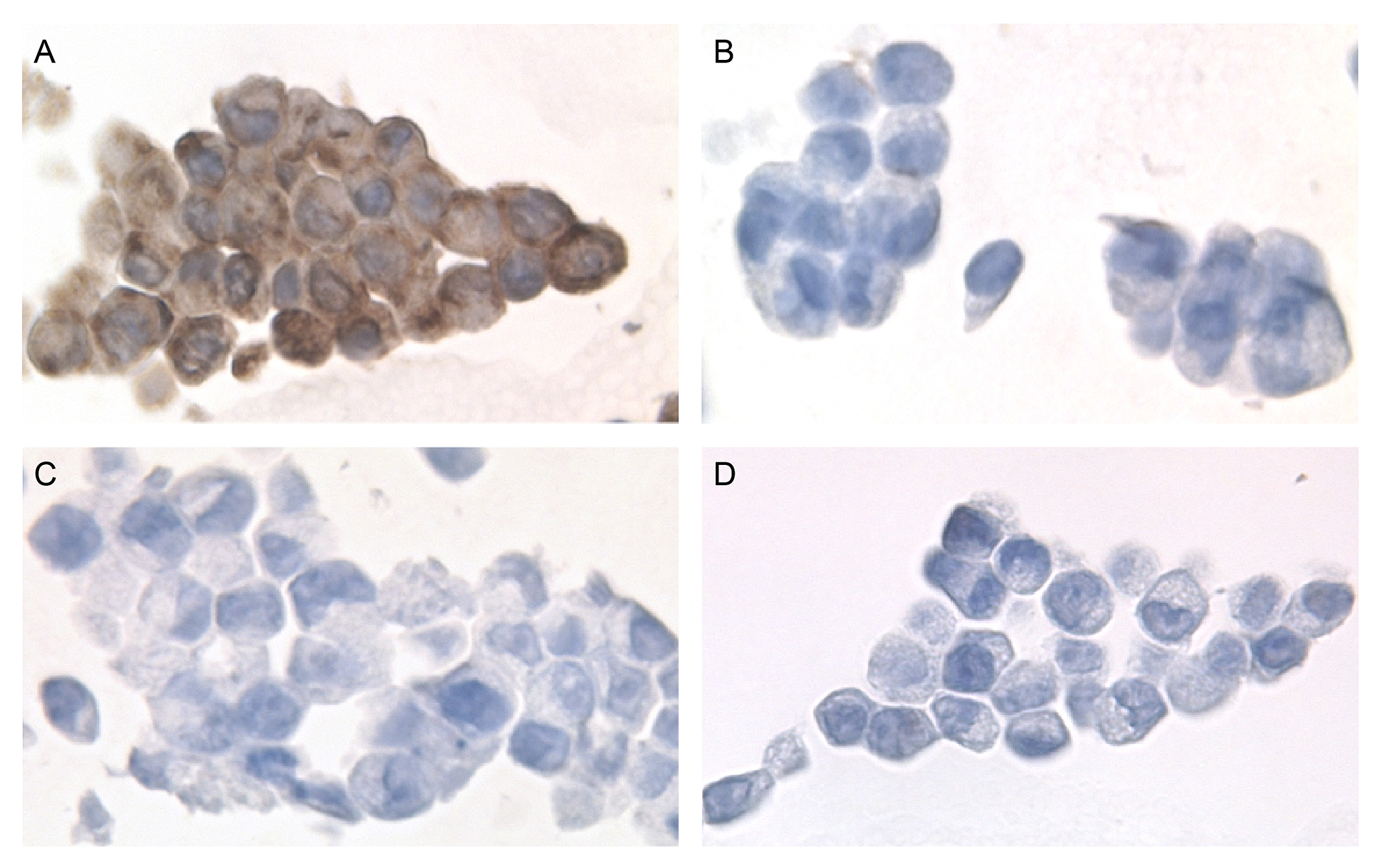

Supplement: Figure S1 — Immunocytochemistry of stably transfected MKN45 cells. LGR5-immunoreactivity in MKN45 gastric cancer cells stably transfected with LGR5 cDNA (LGR5/MKN45) (A) compared to control cells, transfected with the empty vector (B). The lower panel depicts LGR5/MKN45 cells incubated without the primary antibody (C) or after pre-incubation of anti-LGR5-antibody with its immunizing blocking peptide, respectively (D). Original magnifications ×600. (TIF) [file pone.0035486.s002.tif]
